# The sexual brain, genes, and cognition: A machine‐predicted brain sex score explains individual differences in cognitive intelligence and genetic influence in young children

**DOI:** 10.1002/hbm.25888

**Published:** 2022-04-26

**Authors:** Kakyeong Kim, Yoonjung Yoonie Joo, Gun Ahn, Hee‐Hwan Wang, Seo‐Yoon Moon, Hyeonjin Kim, Woo‐Young Ahn, Jiook Cha

**Affiliations:** ^1^ Department of Brain and Cognitive Sciences, College of Natural Sciences Seoul National University Seoul South Korea; ^2^ Institute of Data Science Korea University Seoul South Korea; ^3^ Interdisciplinary Program of Bioengineering, College of Engineering Seoul National University Seoul South Korea; ^4^ College of Liberal Studies Seoul National University Seoul South Korea; ^5^ Department of Psychology, College of Social Sciences Seoul National University Seoul South Korea; ^6^ AI Institute Seoul National University Seoul South Korea

**Keywords:** cognitive performance, gene–brain–cognition pathway, genome‐wide polygenic score, intelligence, machine learning, sex development

## Abstract

Sex impacts the development of the brain and cognition differently across individuals. However, the literature on brain sex dimorphism in humans is mixed. We aim to investigate the biological underpinnings of the individual variability of sexual dimorphism in the brain and its impact on cognitive performance. To this end, we tested whether the individual difference in brain sex would be linked to that in cognitive performance that is influenced by genetic factors in prepubertal children (*N* = 9,658, ages 9–10 years old; the Adolescent Brain Cognitive Development study). To capture the interindividual variability of the brain, we estimated the probability of being male or female based on the brain morphometry and connectivity features using machine learning (herein called a brain sex score). The models accurately classified the biological sex with a test ROC–AUC of 93.32%. As a result, a greater brain sex score correlated significantly with greater intelligence (*p*_fdr_ < .001, $\eta_{p}^{2}$ = .011–.034; adjusted for covariates) and higher cognitive genome‐wide polygenic scores (GPSs) (*p*_fdr_ < .001, $\eta_{p}^{2}$ < .005). Structural equation models revealed that the GPS‐intelligence association was significantly modulated by the brain sex score, such that a brain with a higher maleness score (or a lower femaleness score) mediated a positive GPS effect on intelligence (indirect effects = .006–.009; *p* = .002–.022; sex‐stratified analysis). The finding of the sex modulatory effect on the gene–brain–cognition relationship presents a likely biological pathway to the individual and sex differences in the brain and cognitive performance in preadolescence.

## Graphical abstract

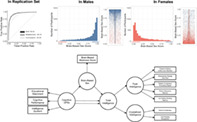

## 1 INTRODUCTION

Sex impacts the development of the brain, cognition, and behaviors. Sex differences in psychological cognitive well‐being, mental, and cognitive health outcomes (Assari, Boyce, & Jovanovic, 2021) and psychopathology (Loso et al., 2021) may originate from sex differences in brain structure and function in children. Several studies attempted to test the sex‐dependent structural and functional variations in the brains of males and females in youth (Satterthwaite et al., 2015; Sepehrband et al., 2018). Human neuroimaging reports sex differences in the total brain volume, cortical thickness, functional, and white matter structural connections (Ingalhalikar et al., 2014; Kaczkurkin, Raznahan, & Satterthwaite, 2019; Kurth, Gaser, & Luders, 2020; Raznahan et al., 2011; Ritchie et al., 2018; Zhang, Dougherty, Baum, White, & Michael, 2018). Others view that the human brains present the mosaics of female and male characteristics with a great extent of individual variability (Joel et al., 2015).

In human literature, reported differences in the brain structure and function do not always map onto those in cognition. This may be related to the fact that the prior research primarily focused on the group differences between sex and thus failed to account for individual variations of the impact of sex on the brain and cognition. Since numerous brain, cognitive, and behavioral traits have extensively overlapping distributions between sex (Joel et al., 2015; Maney, 2016; Vosberg et al., 2020), sex may be considered as a continuous rather than binary factor. Indeed, a recent elegant study shows that the continuum of sex derived from brain or body measures accounts for the individual variability of sex hormone levels and behaviors in adolescents (Vosberg et al., 2020). Therefore, determining how sex affects one's brain, cognition, and individuality is an important research question toward precision neuroscience.

A crucial but understudied factor in the sex differences in brain and cognition is the genetic underpinning. The genetic basis of the brain and cognitive performance, and the relationship with sex may provide new mechanistic insights into the impact of sex on the brain and cognitive performance. Literature shows the heritability and genetic differences across individuals account for between 20 and 70% of the variance in general intelligence (Haworth et al., 2010). Large‐scale genome‐wide association studies (GWAS) have discovered the variants significantly related to cognitive ability in European‐descent populations (Davies et al., 2019; Hill et al., 2019; Savage et al., 2018). Concerning the genetic variants of small effects associated with the heritability of intelligence, a genome‐wide polygenic score (GPS) could provide an overall estimate of the genetic influence on a trait at the individual level (Choi, Mak, & O'Reilly, 2020) and expand its utility to multiethnic populations (Joo et al., 2020; Plym et al., 2021). Indeed, the GPS approach shows the inherited genome‐wide genetic effect accounts for up to 13% of the variance in educational attainment and 7–10% in cognitive performance (Lee et al., 2018). An important outstanding question regarding neurocognition is whether the genetic influence on cognitive performance is related to the impact of sex on the brain and cognition. Testing this relationship may provide an insight into the understanding of the vital biological factors of neurocognitive development.

In this study, we investigate the individual differences in brain sex in a rigorous, unbiased, data‐driven manner leveraging large‐scale multimodal brain imaging of grey matter morphometric and white matter connectomes, together accounting for the key brain processes in prepubertal children. We then test whether the individual differences of the brain sex map onto that of cognitive performance. Finally, we investigate the relationship between brain sex and cognitive performance concerning the genetic influence on cognitive performance. The integrative analysis of brain imaging, cognition, and the GPS in the large pediatric samples provides new and mechanistic insights.

## 2 MATERIALS AND METHODS

### 2.1 ABCD participants

We obtained the study data from the Adolescent Brain Cognitive Development (ABCD) study release 2.0 (http://abcdstudy.org). The ABCD study is the largest longitudinal study of brain development and child health across the United States (Jernigan, Brown, & Dowling, 2018). The ABCD study aims to investigate psychological and neurobiological development trajectories for adolescent mental health (Garavan et al., 2018). Multiethnic children (N = 11,875) with ages of 9–10 years were recruited from 21 research sites composed of 52.3% Caucasians, 20.3% Mexican‐Americans, 14.7% African‐Americans, and 12.5% Asian‐Americans and others based on self‐reported ethnicities. Informed consent and assent forms were collected from all the participants and parents, and/or legal guardians. About 9,658 participants were used for the analysis after quality control (see “Brain Imaging‐Quality Assessment and Control”). Missing values of the income variable (403 missing values in males, 362 missing values in females) were imputed with median values. All experimental protocols were approved by the Seoul National University's institutional review board (IRB).

### 2.2 Cognitive intelligence

We used total intelligence, fluid intelligence, crystallized intelligence composite scores measured by the NIH Toolbox (Luciana et al., 2018). They are based on the following tests: dimensional change card sort test, flanker inhibitory control and attention test, oral reading recognition test, pattern speed test, picture vocabulary test, and list sorting working memory test. The test scores were *z*‐scored and not normalized to age and sex; instead, these were used as covariates in the statistical models. The NIH Toolbox assessment scores of each participant were available from the neurocognitive data of the ABCD release 2.0.

### 2.3 Genome‐wide polygenic scores

We created genome‐wide polygenic scores (GPSs) of three representative cognitive traits: educational attainment, cognitive performance, and intelligence quotient (IQ). The saliva DNA samples were collected for 10,659 participants and genotyped at Rutgers University Cell and DNA Repository (RUCDR) with Affymetrix NIDA Smoke Screen Array. After removing the SNPs with genotype call rate <95%, sample call rate <95%, and minor allele frequency (MAF) <1%, we performed imputation using the Michigan Imputation Server using the 1000 Genome phase3 version5 panel and Eagle v2.4 phasing. Among the imputed variants with INFO score > .3, we additionally removed the inferior SNPs with genotype call rate <95%, sample call rate <95%, rare variants with MAF <1%, and Hardy–Weinberg equilibrium (HWE) *p*‐value <1 × 10^−10^ for an ethnically diverse population. We performed identity‐by‐descent analysis on the pruned data and excluded genetically related individuals closer than first cousin relatedness or grandparent‐grandchild relatedness (phi hat > .18) using PLINK software, which resulted in keeping only one subject from each biological family (Purcell et al., 2007). Then, principal component analysis () of the study samples with 1000 Genome phase3 reference samples was conducted, showing that all the study samples were clustered as admixed American population (super population code AMR), falling inside the mean pairwise Euclidean distance between admixed American samples (Figure S1). Next, GPSs of cognitive phenotypes were computed as the sum of effect allele count (0,1,2) weighted by the effect based on the publicly available summary statistics from the large‐scale GWASs on educational attainment, cognitive performance, and intelligence quotient (IQ) (Lee et al., 2018; Savage et al., 2018). The score estimation with linkage‐disequilibrium clumping was performed using *R* ^2^ > .2 thresholds over 500 kb sliding windows over the reference panel (*p*‐value <1) using PRSice2 software (Euesden, Lewis, & O'Reilly, 2015; Luciana et al., 2018). To evaluate the generalizability of our results to multiethnic populations, we performed sensitivity analyses with European‐ancestry participants (44.04% of total ancestry). We determined genetically the participants of European descent by computing the principal components of individual genotype data and compared those with the European reference samples from HapMap phase 3 (International HapMap 3 Consortium et al., 2010) using the *PLINKQC* R package (Anderson et al., 2010; Chang et al., 2015). For the entire multiethnic analysis, we used self‐reported ethnicity variables as covariates to address potential confounding by population stratification.

### 2.4 Sex hormones

We used saliva salimetrics mean scores (pg/ml) of testosterone and dehydroepiandrosterone (DHEA) available from the curated ABCD dataset. Since estradiol was not collected in males, we excluded the estradiol in the present study. The saliva collection was implemented following Granger, Johnson, Szanton, Out, and Schumann (2012). Participants did not eat or any drink before the saliva collection. There are variations in collection time and the cold chain for the sample protection depending on the ABCD study site (Herting et al., 2020).

### 2.5 Brain imaging–anatomical imaging: T1/T2, Freesurfer 6

T1‐weighted (T1w) and T2‐weighted (T2w) 3D structural MRI were acquired in the ABCD study. The images were processed using the following protocol (Casey et al., 2018; Garavan et al., 2018): To improve geometric accuracy and image intensity reproducibility, the gradient nonlinearity distortion correction method was performed in structural MRI (Jovicich et al., 2006). T2w images were registered to T1w images using mutual information (Wells 3rd, Viola, Atsumi, Nakajima, & Kikinis, 1996). Based on tissue segmentation and sparse spatial smoothing, intensity non‐uniformity was corrected. Then, the data were resampled with 1 mm isotropic voxels into rigid alignment with an atlas brain.

Cortical surface reconstruction was applied using the following procedures: Structural MRI was processed using FreeSurfer v6.0 (https://surfer.nmr.mgh.harvard.edu) for cortical surface reconstruction (Dale, Fischl, & Sereno, 1999), which includes skull‐stripping (Ségonne et al., 2004), white matter segmentation and initial mesh creation (Dale et al., 1999), correction of topological defects, surface optimization (Fischl, Liu, & Dale, 2001; Ségonne, Pacheco, & Fischl, 2007), and nonlinear registration to a spherical surface‐based atlas (Fischl, Sereno, Tootell, & Dale, 1999). For the extraction of brain regions, we used autonomic image segmentation methods using Desikan–Killiany and Destrieux atlas. We extracted 1,086 brain regions, including volumes, surface area, thickness, mean curvature, sulcal depth, and gyrification.

### 2.6 Brain imaging–diffusion spectrum imaging

We used the diffusion spectrum images from the ABCD study preprocessed using the following protocol (Hagler Jr et al., 2019) by the ABCD Data Analysis and Informatics Center (DAIC). Eddy current distortion correction was used with a nonlinear estimation using diffusion gradient orientations and amplitudes to predict the pattern of distortions (Zhuang et al., 2006). Head motion was corrected by registering to images synthesized from tensor fit (Hagler et al., 2009). Diffusion gradients were adjusted for head rotation (Hagler et al., 2009; Leemans & Jones, 2009). We then fitted the diffusion tensor model (Chang, Jones, & Pierpaoli, 2005). B0 distortion was corrected with reversing gradient method (Holland, Kuperman, & Dale, 2010). Gradient nonlinearity distortion correction was applied (Jovicich et al., 2006). We obtained the data by using mutual information of T2‐weighted b0 images to T1w structural images (Wells 3rd et al., 1996). Then, the data were resampled to a standard orientation with 1.77 mm isotropic resolution.

To estimate accurate brain imaging phenotypes, we used individual connectome data. First, we applied MRtrix3 (Tournier et al., 2019) for whole‐brain white matter tracts estimation and individualized connectome generation. For connectivity metrics, we used streamline counts associated with fiber connection strength (Cha et al., 2015, 2016) and fiber integrity. We estimated the spatially varying noise maps and calculated the objective threshold on the eigenvalues for PCA denoising derived from the noise level (Veraart et al., 2016). Then, we performed bias correction with the Advanced Normalization Tools (ANTs) pipeline' N4 algorithm (Tustison et al., 2010). To obtain a connectivity index with a white matter pathway (Ciccarelli et al., 2006), we performed probabilistic tractography by 2nd order integration over fiber orientation distributions (Calamante, Tournier, Jackson, & Connelly, 2010) with random seeding across the brain and target streamline counts of 20 million. These initial tractograms were filtered out preliminary tractograms with spherical‐deconvolution informed filtering (2:1 ratio). With a final streamline count of 10 million, we generated an 84 × 84 whole‐brain connectome matrix for each participant using the T1‐based parcellation and segmentation from FreeSurfer. This pipeline thus ensures that individual participants' connectomes are restricted to their neuroanatomy. Computation was carried out on the supercomputers at Argonne Leadership Computing Facility Theta and Texas Advanced Computing Center Stampede2.

### 2.7 Brain imaging–quality assessment and control

Quality assessment of the ABCD brain imaging data was performed by the ABCD DAIC. The ABCD MRI quality assessment consisted of protocol compliance checking, automated quality control metrics, and manual review of data quality (Hagler Jr et al., 2019). In the first part, the protocol compliance checking included the match between key imaging parameters and expected values for a given scanner, such as voxel size or repetition time. The protocol compliance checking was performed by on‐site FIONA workstations for feedback to scan operators. The presence or absence of the B0 distortion field map series was also checked for diffusion MRI (dMRI). In the second part, automated quality metrics were used. For structural MRI (sMRI), the mean and standard deviation of brain values and spatial SNR was used. For dMRI, mean motion and the number of slices and frames affected by slice dropout due to head motion. Finally, with the combination of automated and manual methods, the DAIC reviewed data quality, such as incorrect acquisition parameters, imaging artifacts, or corrupted data files.

For FreeSurfer cortical surface reconstruction and dMRI preprocessing, we used the quality control results from the ABCD DAIC (Hagler Jr et al., 2019). The DAIC reviewed FreeSurfer cortical surface construction and DTI reconstruction and generated a binary QC score. A sample is deemed unusable when the data has the most severe artifacts or irregularities, results still included in shared tabulated data, and recommended exclusion from group analyses involving cortical, subcortical, and tract‐based ROIs. Reviewers gauged the severity of five types of artifacts for FreeSurfer QC: motion, intensity inhomogeneity, white matter underestimation, pial overestimation, and magnetic susceptibility artifact; and, for DTI, B0 wrapping, image quality based on motion, intensity inhomogeneity, and magnetic susceptibility artifact, full head coverage, registration with T1w image, and registration with FreeSurfer segmentation.

### 2.8 Sex classification with machine learning

We conducted machine learning modeling to classify biological sex, using the brain morphometric and white matter structural connectomic estimates as the input. The features related to the whole brain volume were excluded because of the apparent volume difference between the male and female brains: for example, total intracranial volume, total gray matter volume. We additionally excluded features with a zero‐variance. Finally, we used 988 features for structural MRI and 3,486 features for white matter connectivity. We used an ensemble machine learning pipeline consisting of data loading, feature preprocessing, model and feature tuning for the optimal hyperparameters of light gradient boost machine (von Luxburg et al., 2018), general linear model, and xgboost (Chen & Guestrin, 2016).

**FIGURE 1:**
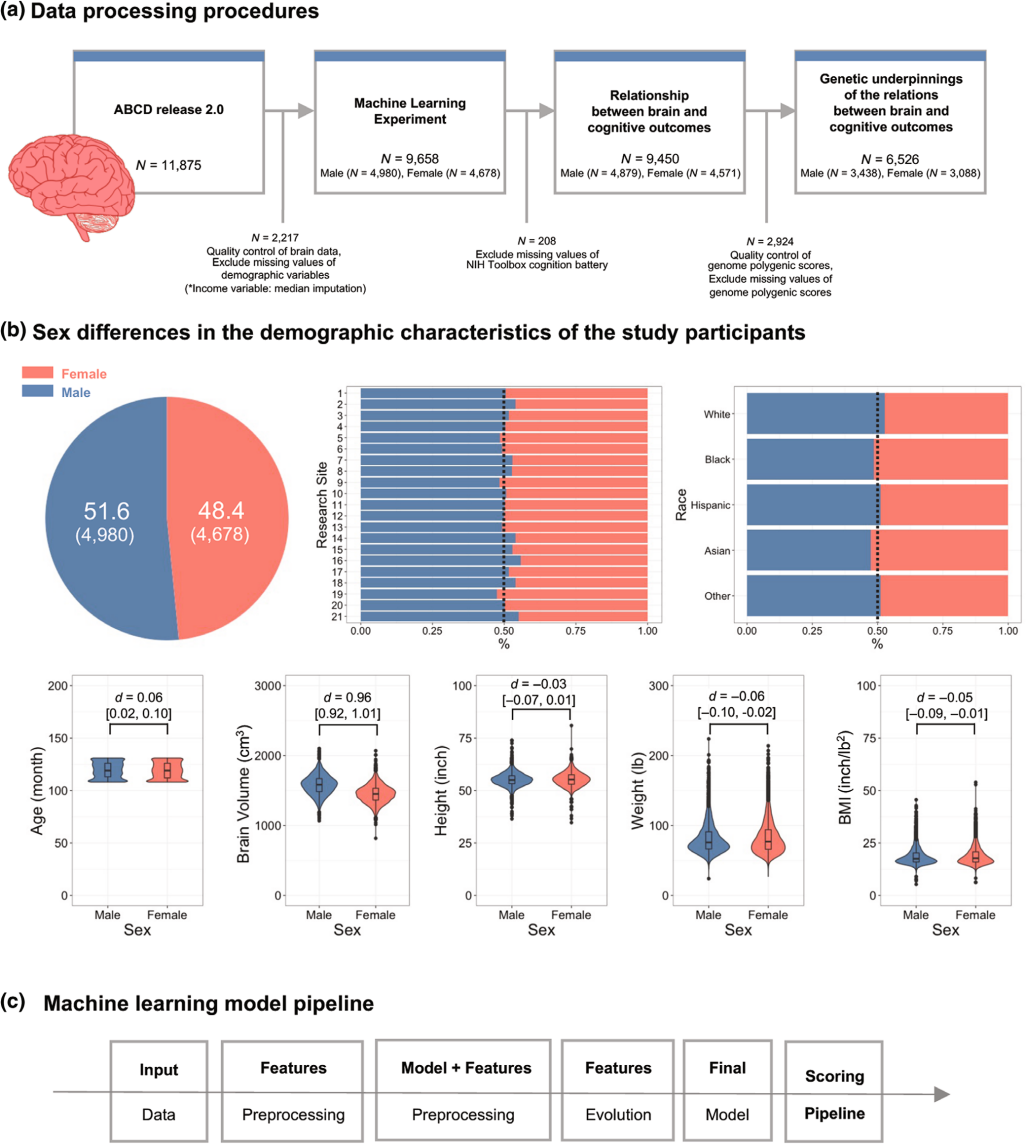
The descriptions of ABCD data and machine learning pipeline. (a) Data processing procedures. (b) Sex differences in the demographic characteristics of the study participants. (c) Machine learning model pipeline

To find the optimal final machine learning model, we performed the following ML pipeline in H2O's Driverless AI package (Hall, Gill, Kurka, & Phan, 2017) (DAI version 1.9.0.6) (Figure 1c). In model and feature tuning, we combined random hyperparameter tuning with feature selection and generation. In each iteration, we updated features using important variables calculated from the previous iteration. Then, the best performing model and features are passed to the feature evolution stage. Feature evolution is the stage that uses a genetic algorithm to find the best set of model parameters and feature transformations to be used in the model. To find the best representation of the data for the final model training, we evaluated the features over iterations and trained and scored 153 models to further evaluate engineered features. This feature evolution approach helps search for the best representation of the data (Whitley, 1994). Then, using those features, 27 models were trained varying the three model algorithms (GLM, GBM, xgboost) and their hyperparameter sets. We finally built a stacked ensemble model with the three best models in discovery sets. A stacking ensemble model is an algorithm that learns how to combine the best prediction models from multiple machine learning models (van der Laan, Polley, & Hubbard, 2007). To test the reproducibility and generalizability, we evaluated the final model's performance in the replication set (20% split of the ABCD). To compare model performance between machine learning and deep learning, we performed deep neural network (DNN), TabNet, to predict the biological sex of the participants. TabNet is a high‐performance and interpretable deep learning architecture with a sequential attention mechanism for feature selection that enables feature interpretability and efficient learning (Figure S2) (Arik & Pfister, 2019).

### 2.9 Machine learning interpretation

To make neural inferences, we applied a machine learning interpretation framework, “K‐Local Interpretable Model‐agnostic Explanation (K‐LIME),” a variant of LIME (Ribeiro, Singh, & Guestrin, 2016), available in H2O's Driverless AI package (Hall et al., 2017). The K‐LIME technique allows both global (i.e., group‐level) and local (i.e., subject‐level) explanations of given machine learning models. K‐LIME generates a single surrogate linear model on the entire training samples for global explanations and multiple local surrogate linear models on samples created from K‐means clusters in the discovery data set for local explanations. The features of K‐means were selected from the variable importance in a random forest surrogate model. To estimate the predictions in a final ensemble model, linear surrogate models were trained. The number of clusters for local explanations were selected based on a grid search, where the *R* ^2^ of predictions was maximized between the final ensemble and all the local K‐LIME models.

### 2.10 Cross‐validation

We split the ABCD data into a discovery (80%) and replication set (20%) stratified by biological sex. Distributions of the covariates (age, race/ethnicity, sites, parental education, marital status, income, height, weight, and BMI) were consistent between the split. We used leave‐one‐site‐out‐cross‐validation (21‐folds) to test the generalizability of model performance across different data collection sites. In addition, we performed random five fold cross‐validation.

### 2.11 General linear model

We tested the relationship between brain‐based sex scores from the ML model and NIH toolbox scales and the cognitive GPSs using a general linear model (GLM). The following covariates were used: age, race/ethnicity, sites, parental education, marital status, income, height, weight, BMI, additionally family ID as a random variable. All the models were fit to the males and females separately. To isolate the effect of brain‐based sex score from that of the total brain volume, we additionally performed Gram‐Schmidt orthogonalization regression. Gram‐Schmidt process orthogonalizes the columns in an inner product space by projecting each successive variable on the previous ones and subtracting (Leon, Björck, & Gander, 2013; Liu, Wang, & Wang, 2018). GLM was then fit to the resultant decorrelated variables.

### 2.12 Structural equation modeling

To test the putative hidden relationship among the cognitive GPSs, brain, and intelligence, we performed the structural equation modeling (SEM) using the lavaan R package (Rosseel, 2012). We defined three latent variables: cognitive GPSs, brain‐based sex, total intelligence from different data sources described above. The GPSs of three representative cognitive traits (educational attainment, cognitive performance, and intelligence quotient) were used as observed variables for cognitive GPSs. Fluid intelligence composite score (flanker inhibitory control and attention, dimensional change card sort, pattern comparison processing speed, picture sequence memory, list sorting working memory) and crystallized intelligence composite score (picture vocabulary, oral reading recognition) from NIH Toolbox assessment data were observed for total intelligence. Parameters were estimated using a bias‐corrected bootstrap method with a 95% confidence interval. We included the following covariates in the models: age, race/ethnicity, sites, parental education, marital status, income, height, weight, and BMI (categorical variables were dummy coded).

## 3 RESULTS

### 3.1 Demographic characteristics

The total brain size showed a significant sexual difference among the demographic variables, with males having a larger total brain size (*p* < .001). Others showed trivial sexual differences (Cohen's *D*s < .06; *p*s < .037; Figure 1b; Table 1).

**TABLE 1:** Demographic characteristics

Demographic data	Mean (*SD*)		*t*/*χ* ^2^ (*p*)	Cohen's *d*/𝜑
	Male	Female		
(*n* = 9,658)	(*n* = 4,980)	(*n* = 4,678)		
Age (months)	119.36 (7.50)	118.91 (7.47)	2.943**	0.06
Height (inches)	55.25 (3.11)	55.35 (3.26)	−1.59	−0.03
Weight (lbs)	81.49 (22.29)	82.91 (23.79)	−3.03**	−0.06
Total brain size (cm^3^)	1,579.61 (140.45)	1,450.47 (127.02)	47.44***	0.96
BMI (inches/lbs^2^)	18.607 (4.01)	18.82 (4.20)	−2.57*	−0.05
Maternal education (highest grade)	16.71 (2.67)	16.69 (2.74)	0.52	0.01
Income	7.33 (2.36)	7.32 (2.36)	0.36	0.01
Ethnicity	White: 2,756 Black: 642 Hispanic: 992 Asian: 93 Other: 497	White: 2,469 Black: 681 Hispanic: 951 Asian: 103 Other: 474	9.34*	0.03
Site	1: 152, 2: 275	1: 149, 2: 235	22.44	0.05
	3: 283, 4: 292	3: 265, 4: 288		
	5: 159, 6: 254	5: 169, 6: 262		
	7: 156, 8: 117	7: 139, 8: 105		
	9: 176, 10: 275	9: 188, 10: 267		
	11: 194, 12: 257	11: 193, 12: 258		
	13: 239, 14: 294	13: 246, 14: 250		
	15: 174, 16: 530	15: 155, 16: 421		
	17: 245, 18: 137	17: 230, 18: 117		
	19: 195, 20: 307	19: 216, 20: 306		
	21: 269	21: 219		
Married	Married: 3,488	Married: 3,210	9.18*	0.03
	Widowed: 38	Widowed: 40		
	Divorced: 460	Divorced: 401		
	Separated: 174	Separated: 184		
	Never married: 539	Never married: 584		
	Living with partner: 281	Living with partner: 259		

*Note*: ***p < .001, **p < .01, *p < .05.

### 3.2 Brain sex classification

**FIGURE 2:**
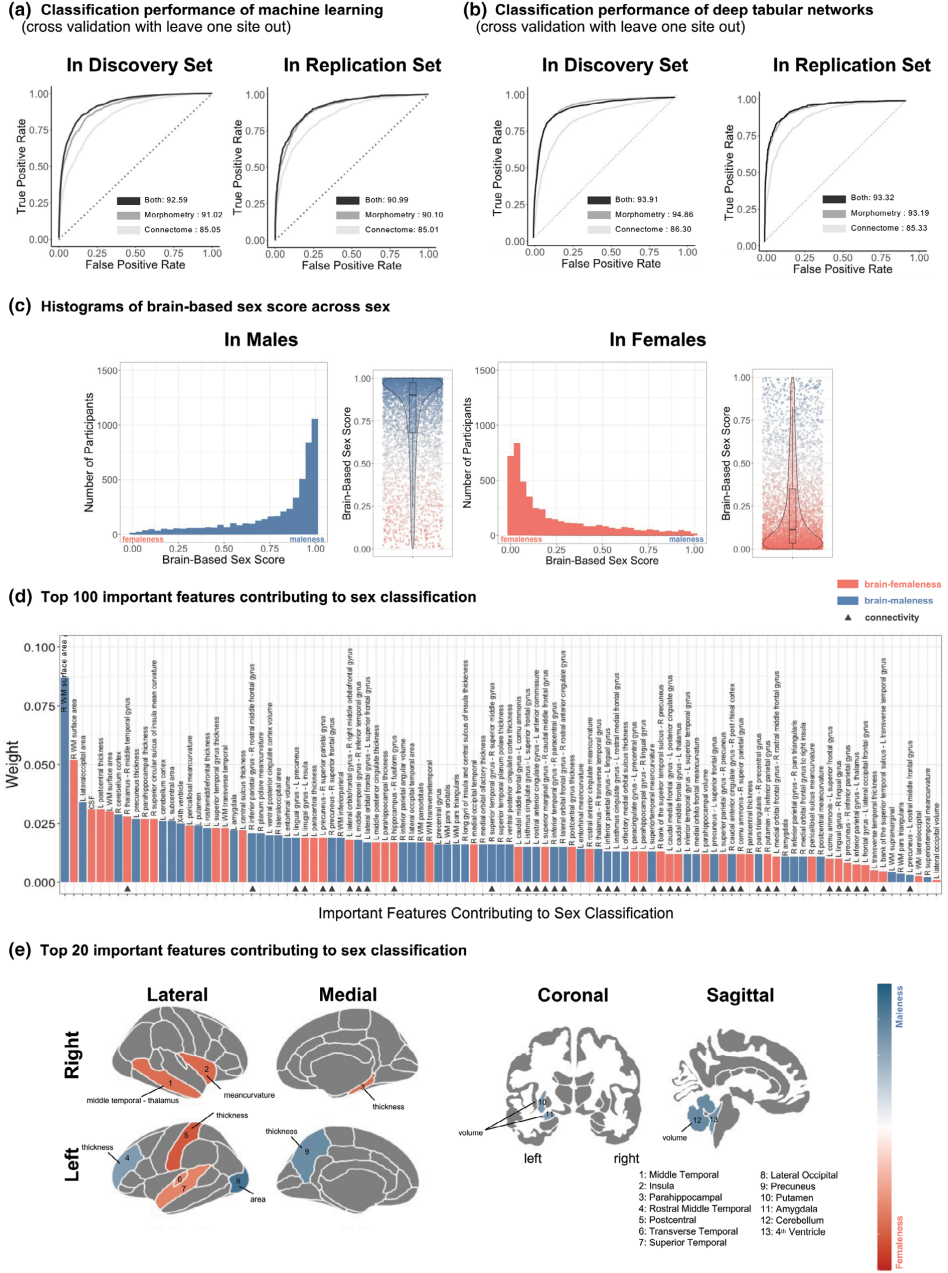
Brain‐based sex score shows individual sex difference in brain. (a) Classification performance of machine learning (leave one site out cross‐validation). (b) Classification performance of deep tabular networks. (c) Histograms of brain‐based sex score across sex. The brain‐based sex score was from the optimized ML model trained on morphometric and structural connectome data. (d) Top 100 important features contributing to sex classification. (e) Top 20 important features contributing to sex classification

We classified sex using machine learning and deep neural networks with morphometric data (including surface area, mean curvature, thickness, and volume) and white matter structure connectomes. Across the cross‐validation methods, combining the morphometric data and white matter structure connectome consistently outperformed the models with morphometric data and white matter structure connectome, respectively (Figure 2a,b; Table S1). In addition, five fold cross‐validation showed slightly better performance than leave‐one‐site‐out‐cross‐validation. The brain‐based sex score from the optimized ML model showed considerable individual differences in both males and females (Figure 2c).

Our machine learning interpretation showed specific brain features contributing to the classification of sex (Figure 2d,e; Table S2). Top 100 important features showed a negative logarithmic curve with a few most important features followed by a number of features with small relative importance (Figure 2d). Among the top 100 features, 41 features were diffusion white matter connectivity (streamline counts), the rest were grey matter morphometric features. The K‐Lime analysis showed that 55% of the features positively contributed to maleness (e.g., an increase in precuneus thickness linked to a greater maleness) and 45% contributed to femaleness (e.g., an increase in postcentral thickness linked to a greater femaleness). This highlights the importance of the spectrum of the brain maleness and femaleness. Furthermore, many of the brain features showed significant, but trivial sex differences (Table S3). That is, each feature alone showed an insufficient statistical power to classify sex, but their aggregated effects (in this case, by means of machine learning) showed a sufficient power to accurately classify sex in a given individual.

### 3.3 Individual differences in brain‐based sex score correlate with cognitive intelligence

**FIGURE 3:**
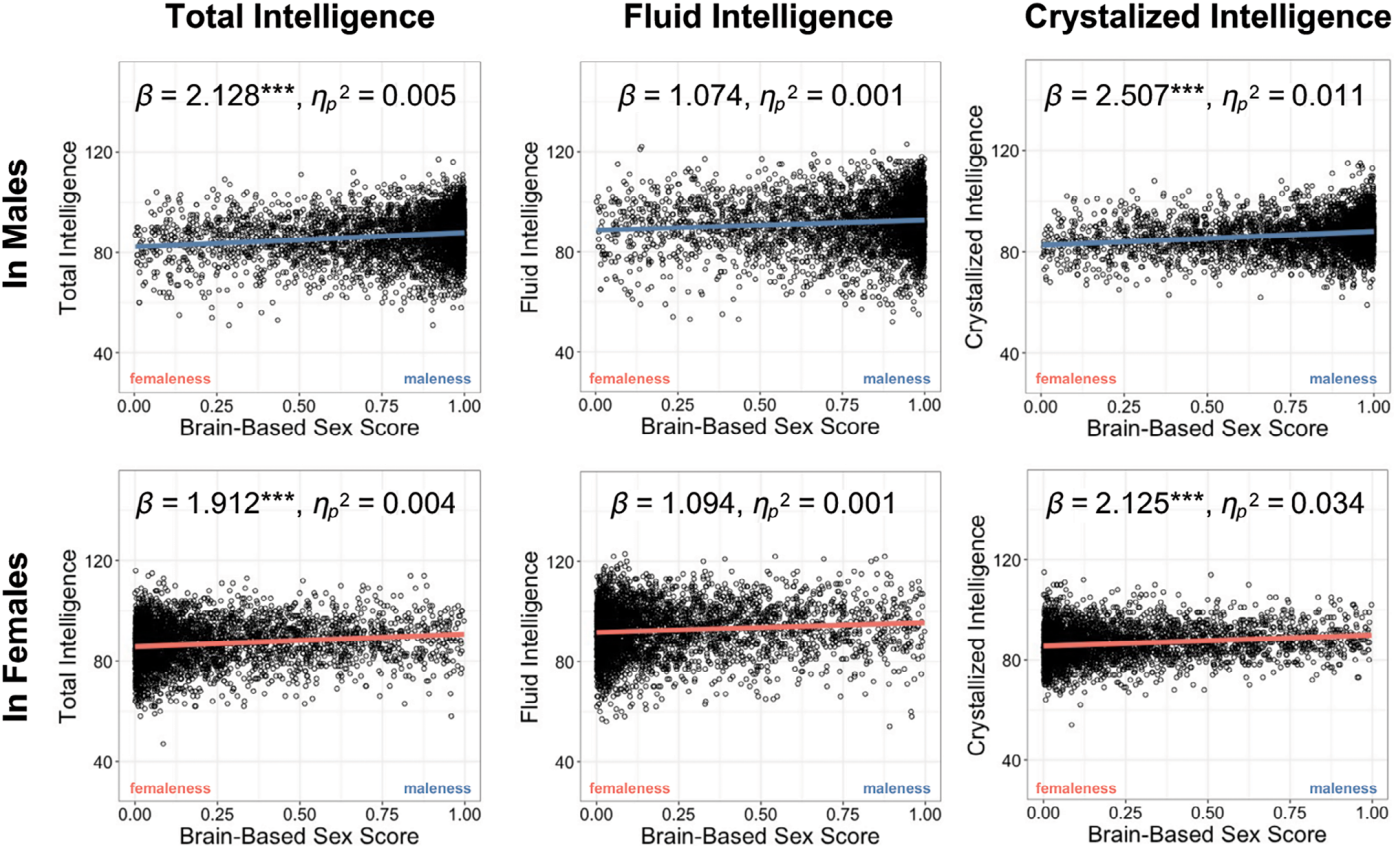
Brain‐based sex score correlates with cognitive intelligence. Associations between brain‐based sex score and cognitive intelligence. Effects adjusted for covariates. ***p < .001, **p < .01, *p < .05

We then tested whether the machine‐predicted brain‐based sex score is linked to cognitive performance in preadolescent children in each sex. As a result, a greater brain‐based maleness score positively correlated with greater cognitive intelligence; reversely, a greater femaleness score negatively correlated with greater cognitive intelligence (Figure 3). Crystallized intelligence showed the most significant positive association with the brain‐based maleness score (Male, *β* = 2.507, *p*_fdr_ < .001, $\eta_{p}^{2}$ = .011; Female, *β* = 2.125, *p*_fdr_ < .001, $\eta_{p}^{2}$ = .034), followed by total intelligence (Male, *β* = 2.128, *p*_fdr_ < .001, $\eta_{p}^{2}$ = .005; Female, *β* = 1.912, *p*_fdr_ < .001, $\eta_{p}^{2}$ = .004). Fluid intelligence showed the smallest, nonsignificant association with brain‐based maleness score (Male, *β* = 1.074, *p*_fdr_ = .094, $\eta_{p}^{2}$ = .001; Female, *β* = 1.094, *p*_fdr_ = .076, $\eta_{p}^{2}$ = .001) (Figure 3, Table S4). Cognitive intelligence showed significant sex differences in intercepts (Table S4); however, the intercepts in our models may not be readily interpretable because our input variables were not mean‐centered.

### 3.4 Individual differences in brain‐based sex score correlate with cognitive GPSs but nonsignificant associations with sex hormones

What is the biological underpinning of the association between brain‐based sex score and intelligence? We tested whether the brain sex‐intelligence association is related to sex hormones and a well‐established genetic influence on cognitive ability (Lee et al., 2018). As a result, sex hormones showed no significant associations with the score of brain sex (maleness) (Male—Testosterone: *p*_fdr_ = .855; DHEA: *p*_fdr_ = .306; Female—Testosterone: *p*_fdr_ = .632; DHEA: *p*_fdr_ = .357) or cognitive abilities (Male—Total Intelligence: *p*_fdr_ = .955 with Testosterone; *p*_fdr_ = .329 with DHEA; Female—Total Intelligence: *p*_fdr_ = .706 with Testosterone; *p*_fdr_ = .954 with DHEA) in prepubertal children (Table S5).

**FIGURE 4:**
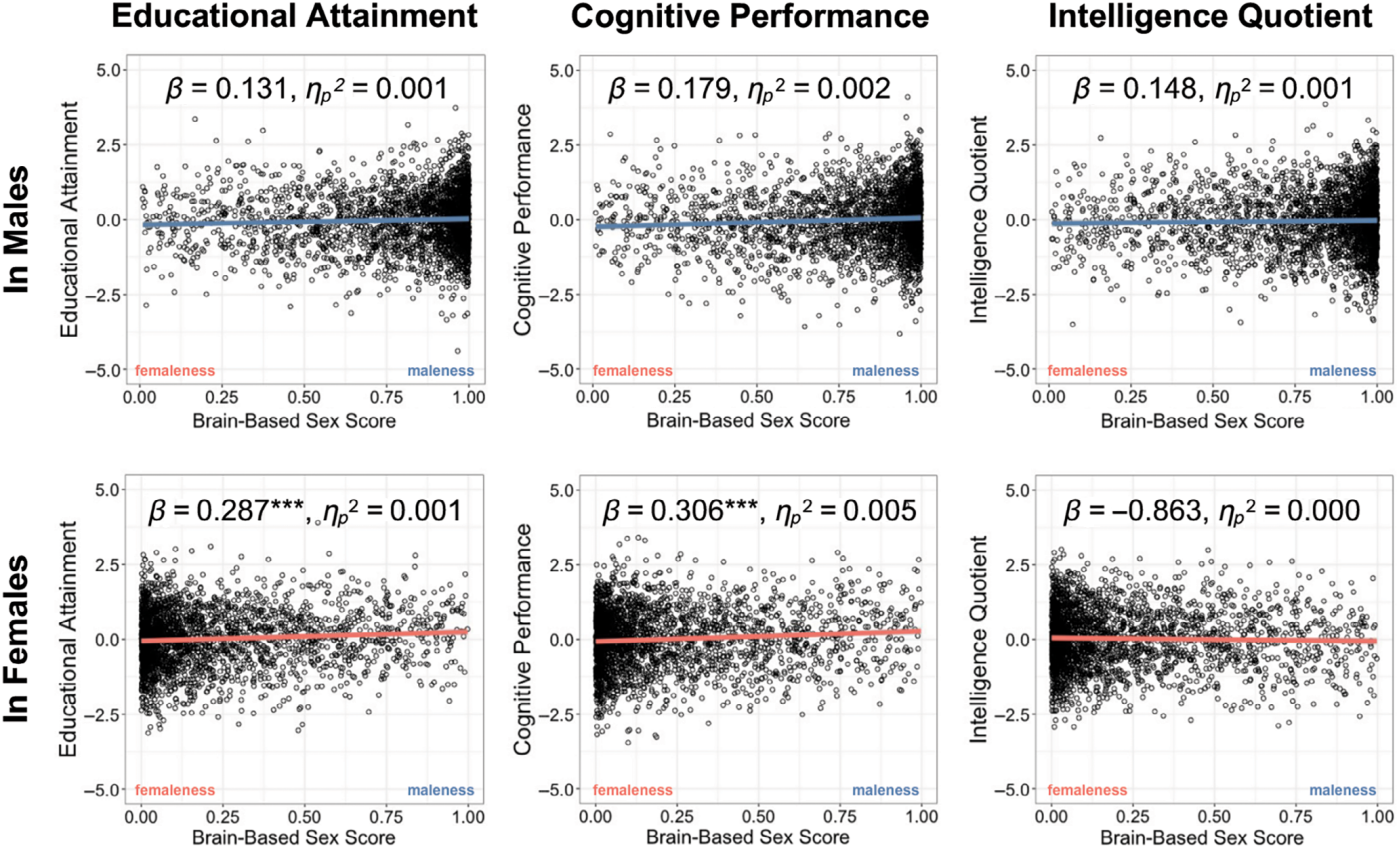
Cognitive GPSs explains genetic underpinnings of the relations between brain and cognitive GPS. Effects adjusted for covariates. ***p < .001, **p < .01, *p < .05

On the other hand, the cognitive GPSs significantly correlated with brain‐based sex (maleness) score and cognitive abilities (Table S6). Of the three cognitive GPSs, the cognitive performance GPS significantly correlated with the brain‐based sex (maleness) score when adjusted for effects of biological sex (*β* = .252, *p*_fdr_ < .001, $\eta_{p}^{2}$ = .004) (Table S7). In the subsequent, sex‐stratified analysis, we found the effect size of this correlation was greater in females than in males (Female—Educational Attainment: *β* = .287, *p*_fdr_ < .001, $\eta_{p}^{2}$ = .001; Cognitive Performance: *β* = .306, *p*_fdr_ < .001, $\eta_{p}^{2}$ = .005; Male—Educational Attainment: *β* = .131, *p*_fdr_ = .082, $\eta_{p}^{2}$ = .001; Cognitive Performance: *β* = 0.179, *p*_fdr_ = .077, $\eta_{p}^{2}$ = 0.002, Intelligence Quotient: *β* = .148, *p*_fdr_ = .082, $\eta_{p}^{2}$ = .001, Figure 4; Table S7). In the analysis of European‐ancestry participants, educational attainment and cognitive performance is significantly related in females (Educational Attainment: *β* = .424, *p*_fdr_ = .001, $\eta_{p}^{2}$ = .014; Cognitive Performance: *β* = .432, *p*_fdr_ = .002, $\eta_{p}^{2}$ = .011; Table S8).

### 3.5 Brain‐based sex score modulates the relationship between cognitive GPSs and cognitive intelligence

**FIGURE 5:**
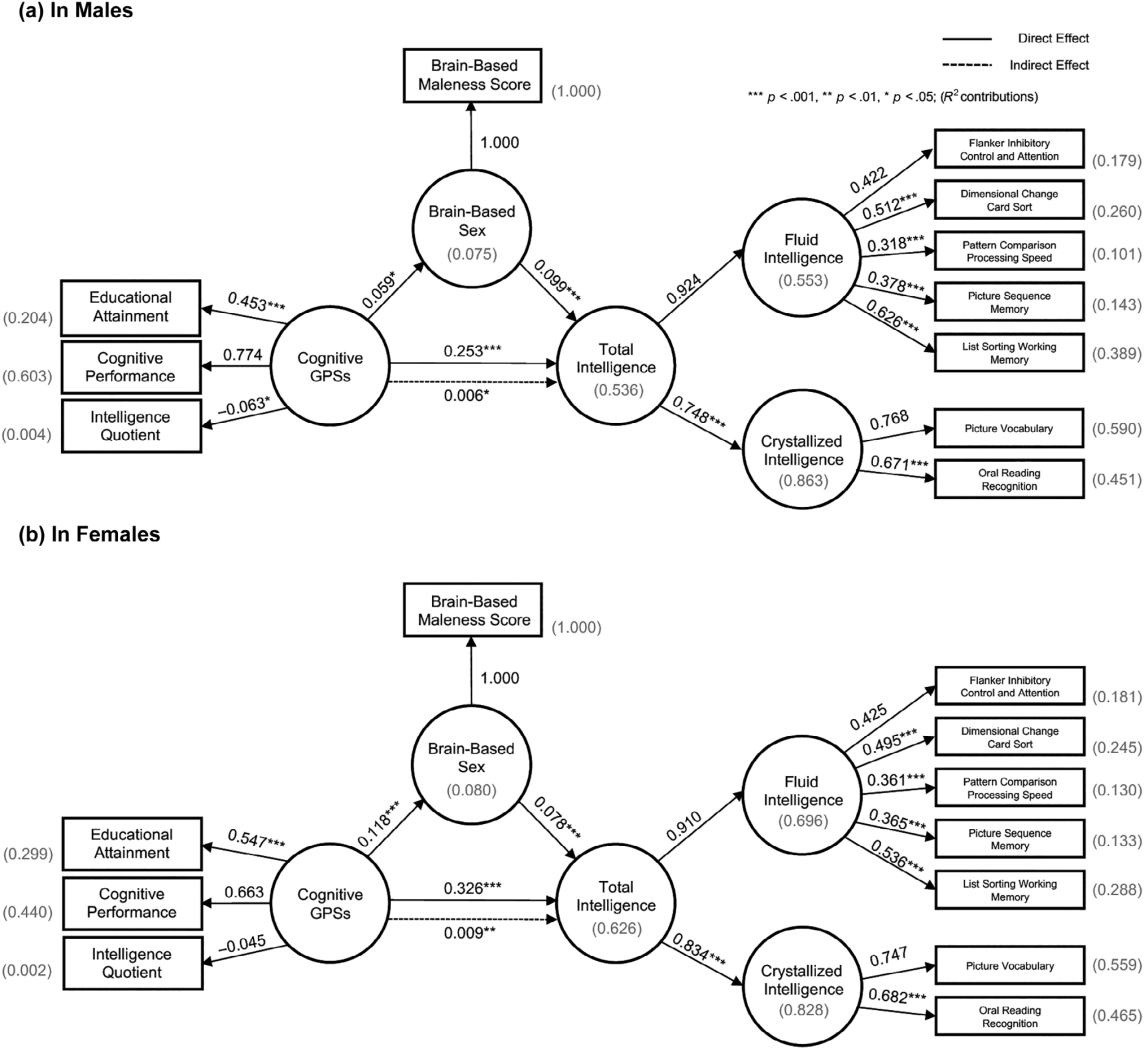
Structural equation modeling of tripartite relationships: Cognitive GPSs–brain sex score‐intelligence. The direct effect is the pathway from the exogenous variable (Cognitive GPSs) to the outcome (Total Intelligence) while controlling for the mediator (Brain‐Based Sex). The indirect effect is the pathway from the exogenous variable (Cognitive GPSs) to the outcome (Total Intelligence) through the mediator (Brain‐Based Sex). (a) Structural equation modeling of tripartite relationships in males. (b) Structural equation modeling of tripartite relationships in females. Standardized weights are shown with statistical significance (bootstrapping). Shown in brackets are explained variances (R^2^). *** < *p* .001

To test the potential hidden relationship among the cognitive GPSs, brain, and intelligence, we performed SEM. In the admixed American participants, sex‐stratified SEM showed acceptable model fits (Comparative Fit Index (CFI)—Male: .921, Female: .940, Root Mean Square Error of Approximation (RMSEA)—Male: .022, Female: .019) (Figure 5). As in the GLMs, the total effect of the cognitive GPSs on intelligence was confirmed significant and positive in both sex (Male—total effect = .253, *p* < .001; Female—total effect = .326, *p* < .001, bias‐corrected percentile method in bootstrap samples). The proportion of the variance for total intelligence explained by cognitive GPSs and brain‐based sex score was *R* ^2^ = .536 in males and *R* ^2^ = .626 in females (*R* ^2^ = .437 in males and *R* ^2^ = .621 in females in European‐ancestry participants). Of note, the brain maleness score partially modulated the cognitive GPS's effect on the total Intelligence in both sexes (Male—indirect effect = .006, *p* = .022; Female—indirect effect = .009, *p* = .002). Note that this means that while the cognitive GPS has a positive direct effect on intelligence in females, a greater brain maleness score (i.e., a lower brain femaleness score) strengthens it. However, when modeling in only European‐ancestry samples, we found no significant indirect modulatory effects of brain sex score (*p*'s > .204) (Figure S6). These results may suggest the modulatory effect of brain sex on the genetic pathway to cognitive performance.

### 3.6 Confounding of the whole brain volume

We found the whole brain volume was significantly correlated with the brain‐based sex score (*p*_fdr_ < .001 in males and females), with the top 10 important features contributing to sex classification (*p*_fdr_ ≤ .008), and with the total Intelligence (*p*_fdr_ < .001). These results together with the literature of the whole brain volume correlating with cognitive intelligence in youth (Reiss, Abrams, Singer, Ross, & Denckla, 1996; Ruigrok et al., 2014; van Leeuwen et al., 2009) led us to the question whether the brain‐based sex score is an epiphenomenon resulting from the two statistically significant patterns: that is, the sex difference in the total brain volume and the correlation between the total brain volume and intelligence. In testing this question, we firstly found that brain‐based sex score better explained cognitive GPSs than the total brain volume did (Brain‐Based Sex Score—Male, *β* = .179, *p*_fdr_ = .077, $\eta_{p}^{2}$ = .002, Female, *β* = .306, *p*_fdr_ < .001, $\eta_{p}^{2}$ = .005 in cognitive performance; Total Brain Volume—Male, *β* = .007, *p*_fdr_ < .001, $\eta_{p}^{2}$ = .006, Female, *β* = .009, *p*_fdr_ < .001, $\eta_{p}^{2}$ = .009 in cognitive performance). Secondly, in an orthogonalized regression model to decorrelate the total brain volume and the brain‐based sex score, we found that the brain‐based sex score independently accounted for the variance of cognitive intelligence (Total Intelligence—Male, *β* = 2,578.335, *p*_fdr_ < .001, $\eta_{p}^{2}$ = 0.008; Female, *β* = 2,978.230, *p*_fdr_ < .001, $\eta_{p}^{2}$ = .025; Fluid Intelligence—Male, *β* = 1,528.188, *p*_fdr_ < .001, $\eta_{p}^{2}$ = .002, Female, *β* = 3,002.295, *p*_fdr_ < .001, $\eta_{p}^{2}$ = .016; Crystallized Intelligence—Male, *β* = 2,853.268, *p*_fdr_ < .001, $\eta_{p}^{2}$ = .016; Female, *β* = 2,043.587, *p*_fdr_ < .001, $\eta_{p}^{2}$ = .019; Table S9). These two results may support that the estimated brain‐based sex score may capture a neurobiological process related to the continuum of brain sex differentiation in each individual above and beyond the total brain volume.

## 4 DISCUSSION

We report the novel relationship between brain sex difference, cognitive performance, and shared genetic influence in an admixed American population of prepubertal children. As trained on the grey matter morphometric and white matter connectomes, our machine learning models showed the accurate classification of sex with over 93.32% ROC–AUC in a replication set. Furthermore, the individual variability of the sexual brain development, indexed by the brain‐based sex score, showed significant correlations with general intelligence and the inherited genetic influence on general intelligence, the cognitive GPSs. Moreover, the SEM showed that the effect of the cognitive GPSs on cognitive outcomes was modulated by the brain sex score significantly in females and with a similar trend in males. Thus, this study indicates the critical role of brain sex in cognitive performance in prepubertal children, influenced by genetic factors, providing a biological account for the individual variability of neurocognition.

Our study departs from the prior literature on sex differences in intelligence in children by showing the role of the continuum of brain sex on cognitive performance. Literature shows that the group sex differences in mind and behaviors, such as hormonal influences (Vuoksimaa, Kaprio, Eriksson, & Rose, 2012), brain differences (Ostatníková et al., 2010), cultural influences (Penner & Paret, 2008), gender stereotypes (Stoet & Geary, 2012), and biopsychosocial interactions (Haier, Karama, Leyba, & Jung, 2009; Miller & Halpern, 2014). In intelligence, however, literature shows mixed findings of sex differences (Dykiert, Gale, & Deary, 2009). Some show that males have advantages (Irwing & Lynn, 2005; Jackson & Philippe Rushton, 2006; van der Linden, van der Linden, Dunkel, & Madison, 2017) in general intelligence over females, while others show females have advantages over males (Keith, Reynolds, Patel, & Ridley, 2008). These mixed findings may allude to large individual variability in intelligence within sex. Indeed, a recent seminal study shows the biological underpinnings of the individual variability in behavioral phenotypes in adolescents (Vosberg et al., 2020). This study presents an estimate of the continuum of sex based on the brain and body traits, which predicts within each sex the individual variability in sex hormones, personality traits, and internalizing–externalizing behaviors. In line with this, our study further demonstrates the utility of multimodal brain imaging combined with machine learning in estimating an individual status of brain sex. For example, our method permitted the accurate estimation of an individual's developmental status of the brain sex and revealed that the brain sex estimates varied across individuals even within the narrow age range. The discovery of the correlation of the brain sex variability with the genetic and cognitive variables further reflects that this novel estimate may represent a critical neurobiological process.

Another pattern to note is the greater association of crystallized intelligence (the ability that is acquired throughout life: i.e., knowledge, facts, and skills) with the brain‐based sex, as well as GPSs for cognitive capacity, compared with fluid intelligence (the ability to reason and solve problems in novel situations; a trend towards significance). These findings are partially in line with prior genetic research showing that crystallized intelligence is greatly associated with genetic influence than fluid intelligence (Christoforou et al., 2014; Genç et al., 2021). Furthermore, since learning attitude (i.e., reading books) may be genetically inherited (Krapohl et al., 2014; Olson, Vernon, Harris, & Jang, 2001), it adds to the genetic propensity of crystallized intelligence. Taken together, these empirical findings including ours may challenge the historical conceptualization that fluid intelligence may be more driven by genes and crystallized intelligence by the environment (Cattell, 1971).

Our structural equation models show the potential relationships among the genes, brain sex, and cognition. The results indicate that a higher brain maleness score (a lower femaleness score) positively modulates the positive effect of the cognitive GPS on general intelligence significantly in both sexes. Considering that the modulatory effect remains significant after controlling for several potential confounding factors of the brain and cognitive performance, this GPS‐brain sex‐intelligence pathway has a significant statistical association. These results thus suggest the novel role of brain sex in children, linking the genetic influence to cognitive performance.

Then, what is the biological account of the modulatory effects of the brain sex on the genetic influence on cognitive performance: that is, positive toward maleness and negative toward femaleness? Literature shows that sex chromosomes play a crucial role in cognitive performance (Bender, Puck, Salbenblatt, & Robinson, 1990; Hong & Reiss, 2014; Warling et al., 2020). However, since we did not include the sex chromosomes when constructing the GPSs (following the common practice of the GWAS designs to boost statistical power), it might not fully explain the differences in the mediation effects across sex. Alternatively, we speculate that the different expression patterns of autosomal variants across sex (Boraska et al., 2012; Wijchers & Festenstein, 2011; Zuo et al., 2015) may account for the modulatory effects of sex. Indeed, in line with this speculation, recent literature highlights sex differences in brain transcriptomes related to schizophrenia and alcohol effects (Hitzemann et al., 2021; Hoffman et al., 2022). Future research may test the association between sex differences in genetic expression in the brain and neurocognitive development.

Note that only females showed a significant correlation between brain‐based sex score and cognitive GPSs, whereas males showed a marginally significant correlation after correction for multiple comparisons. We think this should not be interpreted as the female‐only effect of the cognitive GPSs in the brain sex development. Rather, it should be noted that their effect sizes were similar across sex (in educational attainment GPS) and the models combining males and females showed the significant correlations of the brain‐based sex score and cognitive GPSs (in educational attainment and cognitive performance GPSs). Furthermore, regardless of the modulatory effects of sex, in both females and males, the influence of the cognitive GPSs on cognition was positive. This is in line with the literature in adults (Lee et al., 2018; Savage et al., 2018). Taken together, we think that the genetic underpinnings of cognitive development might be related to sex differentiation in the brain. Therefore, our integrative analysis reveals the subtle relationships among sex, genes, brains, and cognition, otherwise undetectable. We suggest this is a novel biological pathway to individual differences in brain sex. It may be interesting to test whether this pathway is related to epigenetic effects of environmental factors, such as early life stress.

This study confirms that biological sex can be classified accurately based on morphometric and white matter connectivity. A recent study with ABCD data show that the biological sex was classified with 89.6% accuracy in the replication set using a deep neural network trained on ABCD T1‐weighted structural MRI (Adeli et al., 2020). Our study extends this prior work by showing the additive classification performance increase with the diffusion white matter connectomes. This performance increase perhaps presents that the multimodal MRI effectively accounts for the heterogeneous developmental trajectories of grey and white matter (Giedd et al., 1999). It further shows the importance of the multimodal MRI approach in accurate delineation of brain development status.

Our brain features exclude the total volumes of the brain, grey and white matter, of which the sex differences have been reported (Ruigrok et al., 2014). Though the whole brain volume difference between sexes may be a biological aspect, we reasoned that the measures of gross anatomy would confound the brain–cognition relationship. Therefore, beyond the sex difference in the gross anatomy, this study shows that the patterns of the grey matter and white matter fibers are associated with the continuum of brain sex.

In testing the relationships among the brain sex, cognitive ability, and the genetic influence on cognitive ability, we focused on the cognitive GPSs. However, our discovery of the significant tripartite correlation among the brain‐based sex score, total brain volume, and intelligence may lead to a question whether the genetic underpinning of cognitive ability is related to that of the total brain size. Indeed, a recent GWAS meta‐analysis reveals an overlap of GWAS hits between cognitive intelligence and brain size in 5 genomic loci (Jansen et al., 2020). We hope that future research may test the moderation effect of sex on the genetic influence on brain size and its impact on cognitive intelligence.

In our study, we found no significant relationship among our key variables with salivary measures of sex hormones. Given the prepubertal stages of the participants, the negative statistical findings may reflect that the gene–brain sex–cognition relationship is not significantly related to the effects of sex hormones. Literature shows a complex relationship between the level of sex hormones and cognitive intelligence (Castanho et al., 2014; Gurvich, Hoy, Thomas, & Kulkarni, 2018). Though different sex hormonal levels across the sexes are observed from the prepubertal ages (Courant et al., 2010), the actual effect of the sex hormones on cognitive intelligence (or its modulation) may not appear until puberty (Shangguan & Shi, 2009).

This study shows a novel relationship among genetic factors, brain sex, and cognitive intelligence. The link between genome‐wide factors and cognitive ability has been shown in previous studies. Cognitive GPSs account for general cognitive ability up to 3.5% in pre‐adolescence children (Allegrini et al., 2019), 11% of the variance in general intelligence, and 16% of the variance in educational achievement in adolescents (Selzam et al., 2017). Extending this literature, our study shows that an individual's degree of brain sex may modulate the impact of the genetic factor on cognitive intelligence. Since this modulatory effect is positive toward brain maleness and negative toward brain femaleness, it adds another source of sex and individual variability in intelligence. This inference also presents the benefit of using the brain data as an endophenotype in assessing the genotype–phenotype association (Glahn, Thompson, & Blangero, 2007). Taken together, brain sex is linked to the inherited genetic influence of cognition, accounting for a novel pathway to the individual difference in cognitive intelligence in preadolescence.

In contrast to the multiethnic participants, the SEM in the European‐ancestry participants only showed nonsignificant indirect effects of brain sex score. The discrepant results may not be easily reconciled. It should be noted that the cross‐ethnic transferability of our cognitive GPS based on the European‐ancestry GWAS remains to be validated. However, our cognitive GPS was rigorously adjusted for the potential ethnic confounding. Our result of the significant modulatory effects in the admixed American participants needs to be interpreted with caution.

This study shows the novel relationships among brain sex, cognition, and cognitive GPSs. The brain sex score based on grey matter morphometric and white matter connectivity may represent a neurodevelopmental process in preadolescence related to the inherited genetic influence on cognitive intelligence and unrelated to sex hormonal levels. This study thus provides a novel framework for future research in neurocognitive development and mental disorders.

## AUTHOR CONTRIBUTIONS

**Kakyeong Kim:** Conceived and designed the experiments; performed the experiments; analyzed the data; interpreted the data; contributed materials/analysis tools; wrote the article; critical revision of the manuscript for important intellectual content. **Yoonjung Yoonie Joo:** Interpreted the data; contributed materials/analysis tools; wrote the article; critical revision of the manuscript for important intellectual content. **Gun Ahn:** Contributed materials/analysis tools. **Hee‐Hwan Wang:** Contributed materials/analysis tools. **Seo‐Yoon Moon:** Contributed materials/analysis tools. **Hyeonjin Kim:** Critical revision of the manuscript for important intellectual content. **Woo‐Young Ahn:** Critical revision of the manuscript for important intellectual content. **Jiook Cha:** Conceived and designed the experiments; interpreted the data; wrote the article; critical revision of the manuscript for important intellectual content; obtained funding; study supervision.

## Supporting information


**Figure S1**. PCA biplots of the genotype data of the study samples with 1000 Genomes phase3 reference samples.
**Figure S2**. Deep neural networks (DNN) architecture.
**Figure S3**. Cognitive intelligence across sex and brain‐based sex group.
**Figure S4**. Characteristics of hormones across sex and brain‐based sex.
**Figure S5**. Characteristics of cognitive GPSs across sex and brain‐based sex.
**Figure S6**. Structural equation modeling of tripartite relationships: cognitive GPSs ‐ brain‐based sex score ‐ cognitive intelligence in European‐ancestry individuals.
**Figure S7**. Structural equation modeling of tripartite relationships: cognitive GPSs ‐ brain size ‐ cognitive intelligence.

## Data Availability

The ABCD data can be accessed via the NIMH Data Archive (https://nda.nih.gov/) and the code used in this study are available for reproducibility (https://github.com/Transconnectome/cGPS-BrainSex-Intelligence).
