# Supplementary material for: The sexual brain, genes, and cognition: A machine‐predicted brain sex score explains individual differences in cognitive intelligence and genetic influence in young children
Source: Hum Brain Mapp. 2022 Apr 26;43(12):3857–72. doi: 10.1002/hbm.25888 (PMC9294341; doi:10.1002/hbm.25888)
Supplement: Supplementary file 1 — Figure S1. PCA biplots of the genotype data of the study samples with 1000 Genomes phase3 reference samples. Figure S2. Deep neural networks (DNN) architecture. Figure S3. Cognitive intelligence across sex and brain‐based sex group. Figure S4. Characteristics of hormones across sex and brain‐based sex. Figure S5. Characteristics of cognitive GPSs across sex and brain‐based sex. Figure S6. Structural equation modeling of tripartite relationships: cognitive GPSs ‐ brain‐based sex score ‐ cognitive intelligence in European‐ancestry individuals. Figure S7. Structural equation modeling of tripartite relationships: cognitive GPSs ‐ brain size ‐ cognitive intelligence. [file HBM-43-3857-s001.pdf]

Supplementary Materials

The sexual brain, genes, and cognition: A machine-predicted brain sex score explains individual differences in cognitive intelligence and genetic influence in young children.

Kakyeong Kim, Yoonjung Yoonie Joo, Gun Ahn, Hee-Hwan Wang, Seo-Yoon Moon, Hyeonjin Kim, Woo-Young Ahn, Jiook Cha

Supplementary Table 1 – 9

Supplementary Figure 1 – 7

Supplementary Table 1. Classification performance of machine learning and deep tabular neural networks

	**Automated machine learning** (cross-validation with leave one site out)		**Deep tabular neural networks** (cross-validation with leave one site out)		**Automated machine learning** (5-cross validation)	
	**Discovery ROC-AUC**	**Replication ROC-AUC**	**Discovery ROC-AUC**	**Replication ROC-AUC**	**Discovery ROC-AUC**	**Replication ROC-AUC**
**Morphometric only**	91.02 (89.21-92.83)	90.10 (88.29-91.91)	94.86 (89.59-98.23)	93.19 (88.69-97.94)	90.88 (90.17-91.59)	90.79 (90.04-91.54)
**Structural connectome only**	85.05 (80.85-89.25)	85.01 (80.81-89.21)	86.30 (75.80-97.86)	85.33 (82.97-87.69)	86.15 (85.23-87.07)	85.46 (84.54-86.38)
**Morphometric and structural connectome both**	92.59 (90.81-94.37)	90.99 (89.21-92.77)	93.91 (89,52-100.00)	93.32 (91.56-94.81)	92.64 (91.74-93.54)	91.23 (90.33-92.13)

Supplementary Table 2. Important features contributing sex classification in morphometric and structural connectome data

Rank	Features contributing to brain-femaleness		Features contributing to brain-maleness	
	Variable	Weight	Variable	Weight
**1**	R white matter surface area	0.052	R white matter surface area	0.087
**2**	Cerebrospinal Fluid (CSF)	0.031	L lateral occipital area	0.034
**3**	L postcentral thickness	0.031	R cerebellum cortex volume	0.029
**4**	L white matter surface area	0.030	L precuneus thickness	0.027
**5**	R thalamus - R middle temporal gyrus	0.028	L cerebellum cortex volume	0.026
**6**	R superior circular sulcus of insula mean curvature	0.027	L subcentral gyrus and sulcus area	0.026
**7**	R parahippocampal thickness	0.027	Fourth ventricle	0.025
**8**	L pericallosal sulcus mean curvature	0.024	L putamen volume	0.024
**9**	L superior temporal gyrus	0.023	L rostral middle frontal thickness	0.023
**10**	L WM transverse temporal gyrus	0.023	L amygdala volume	0.022

Supplementary Table 3. General linear model between top important features and total brain size Effects adjusted for covariates. *** P<0.001, ** P<0.01, * P<0.05

Top 10 features contributing to sex classification	Total brain size in male group					Total brain size in female group				
	β	SE	Y_{int}	P_{fdr}	η_p^2	β	SE	Y_{int}	P_{fdr}	η_p^2
R white matter surface area	0.000	0.000	-8.370	0.000	0.691	0.000	0.000	-7.782	0.000	0.685
Cerebrospinal Fluid (CSF)	0.000	0.000	-5.356	0.000	0.104	0.000	0.000	-4.707	0.000	0.115
R white matter surface area	0.000	0.000	-8.368	0.000	0.691	0.000	0.000	-7.785	0.000	0.685
L postcentral thickness	0.000	0.000	0.134	0.000	0.052	0.000	0.000	1.641	0.000	0.042
L lateral occipital area	0.000	0.000	-4.795	0.000	0.163	0.000	0.000	-4.351	0.000	0.162
R cerebellum cortex volume	0.000	0.000	-9.421	0.000	0.318	0.000	0.000	-7.179	0.000	0.328
L precuneus thickness	0.000	0.000	2.719	0.126	0.006	0.000	0.000	2.574	0.001	0.000
L white matter surface area	0.000	0.000	-8.411	0.000	0.689	0.000	0.000	-7.877	0.000	0.688
R thalamus – R middle temporal gyrus	0.000	0.000	0.612	0.089	0.004	0.000	0.000	2.660	0.008	0.011
R superior circular sulcus of insula mean curvature	0.000	0.000	0.691	0.000	0.003	0.000	0.000	1.246	0.000	0.003

Supplementary Table 4. General linear model between brain-based sex score and cognitive intelligence Effects adjusted for covariates. *** P<0.001, ** P<0.01, * P<0.05

Brain-based sex score	Brain-based sex score In both group					Brain-based sex score in male group					Brain-based sex score in female group				
	β	SE	Y_{int}	P_{fdr}	η_p^2	β	SE	Y_{int}	P_{fdr}	η_p^2	β	SE	Y_{int}	P_{fdr}	η_p^2
Total intelligence	1.999	0.348	37.351	0.000	0.004	2.128	0.502	52.195	0.000	0.005	1.912	0.484	24.487	0.000	0.004
Fluid intelligence	1.076	0.444	48.823	0.015	0.001	1.074	0.641	72.012	0.094	0.001	1.094	0.616	26.645	0.076	0.001
Crystallized intelligence	2.254	0.270	46.030	0.000	0.009	2.507	0.386	48.053	0.000	0.011	2.125	0.383	47.396	0.000	0.034

Supplementary Table 5. General linear model between sex hormones and brain-based sex score or cognitive Intelligence Effects adjusted for covariates. *** P<0.001, ** P<0.01, * P<0.05

General linear model between sex hormones and brain-based sex score															
Hormones	Brain-based sex score in both group					Brain-based sex score in male group					Brain-based sex score in female group				
	β	SE	Y_{int}	P_{fdr}	η_p^2	β	SE	Y_{int}	P_{fdr}	η_p^2	β	SE	Y_{int}	P_{fdr}	η_p^2
Tes tosterone	-0.546	1.324	-53.363	0.680	0.000	-0.342	1.868	-63.440	0.855	0.000	-0.915	1.911	-27.556	0.632	0.000
DHEA	-4.893	3.801	-168.894	0.199	0.001	-5.035	4.911	-146.40	0.306	0.001	-5.502	5.969	-116.767	0.357	0.003
General linear model between testosterone and cognitive intelligence															
Tes tosterone	Brain-based sex score in both group					Brain-based sex score in male group					Brain-based sex score in female group				
	β	SE	Y_{int}	P_{fdr}	η_p^2	β	SE	Y_{int}	P_{fdr}	η_p^2	β	SE	Y_{int}	P_{fdr}	η_p^2
Total intelligence	-0.003	0.010	47.210	0.892	0.000	-0.002	0.014	62.170	0.955	0.000	-0.010	0.014	25.810	0.872	0.000
Fluid intelligence	0.002	0.012	56.610	0.892	0.000	-0.004	0.017	79.890	0.955	0.001	0.003	0.017	27.050	0.885	0.000
Crystallized intelligence	-0.006	0.008	55.120	0.892	0.003	0.001	0.011	55.675	0.955	0.000	-0.013	0.011	49.050	0.706	0.001

General linear model between DHEA and cognitive intelligence															
DHEA	Brain-based sex score in both group					Brain-based sex score in male group					Brain-based sex score in female group				
	β	SE	Y_{int}	P_{fdr}	η_p^2	β	SE	Y_{int}	P_{fdr}	η_p^2	β	SE	Y_{int}	P_{fdr}	η_p^2
Total intelligence	0.004	0.003	48.080	0.288	0.001	0.006	0.005	63.200	0.329	0.001	0.002	0.004	26.200	0.954	0.001
Fluid intelligence	0.005	0.004	57.420	0.288	0.001	0.005	0.007	80.810	0.458	0.000	0.004	0.005	27.530	0.954	0.001
Crystallized intelligence	0.002	0.003	55.780	0.443	0.000	0.006	0.004	57.590	0.329	0.002	--0.001	0.003	49.240	0.954	0.000

Supplementary Table 6. General linear model between cognitive GPS and cognitive intelligence Effects adjusted for covariates. *** P<0.001, ** P<0.01, * P<0.05

Total intelligence	Cognitive GPSs in both group					Cognitive GPSs in male group					Cognitive GPSs in female group				
	β	SE	Y_{int}	P_{fdr}	η_p^2	β	SE	Y_{int}	P_{fdr}	η_p^2	β	SE	Y_{int}	P_{fdr}	η_p^2
Educational attainment	0.772	0.108	47.472	0.000	0.026	0.501	0.154	57.511	0.002	0.004	1.069	0.150	32.410	0.000	0.020
Cognitive performance	1.171	0.100	47.851	0.000	0.026	1.184	0.143	59.484	0.000	0.025	1.179	0.138	31.350	0.000	0.029
Intelligence quotient	-0.086	0.100	45.620	0.387	0.001	-0.168	0.140	56.677	0.233	0.003	-0.005	0.143	29.640	0.971	0.000
Fluid intelligence	Cognitive GPSs in both group					Cognitive GPSs in male group					Cognitive GPSs in female group				
	β	SE	Y_{int}	P_{fdr}	η_p^2	β	SE	Y_{int}	P_{fdr}	η_p^2	β	SE	Y_{int}	P_{fdr}	η_p^2
Educational attainment	0.420	0.136	60.341	0.003	0.002	0.100	0.196	73.710	0.609	0.000	0.775	0.188	41.282	0.000	0.007
Cognitive performance	0.842	0.126	60.951	0.000	0.009	0.890	0.183	75.886	0.000	0.009	0.817	0.174	40.400	0.000	0.009
Intelligence quotient	-0.047	0.126	59.334	0.710	0.000	-0.166	0.178	73.877	0.528	0.002	-0.005	0.143	29.640	0.971	0.000
Crystallized intelligence	Cognitive GPSs in both group					Cognitive GPSs in male group					Cognitive GPSs in female group				
	β	SE	Y_{int}	P_{fdr}	η_p^2	β	SE	Y_{int}	P_{fdr}	η_p^2	β	SE	Y_{int}	P_{fdr}	η_p^2
Educational attainment	0.879	0.086	52.299	0.000	0.020	0.734	0.120	55.781	0.000	0.014	1.031	0.122	46.000	0.000	0.029
Cognitive performance	1.131	0.079	52.332	0.000	0.038	1.082	0.112	56.892	0.000	0.034	1.193	0.112	45.060	0.000	0.045
Intelligence quotient	-0.099	0.080	50.191	0.216	0.000	-0.106	0.110	54.204	0.335	0.003	-0.094	0.117	43.250	0.419	0.000

Supplementary Table 7. General linear model between brain-based sex score and Cognitive GPS Effects adjusted for covariates. *** P<0.001, ** P<0.01, * P<0.05

Cognitive GPS	Brain-based sex score in both group					Brain-based sex score in male group					Brain-based sex score in female group				
	β	SE	Y_{int}	P_{fdr}	η_p^2	β	SE	Y_{int}	P_{fdr}	η_p^2	β	SE	Y_{int}	P_{fdr}	η_p^2
Educational attainment	0.213	0.054	-2.394	0.000	0.003	0.131	0.075	-2.364	0.082	0.001	0.287	0.077	-2.327	0.000	0.001
Cognitive performance	0.252	0.058	-1.874	0.000	0.004	0.179	0.080	-2.660	0.077	0.002	0.306	0.083	-9.759	0.000	0.005
Intelligence quotient	0.028	0.058	-0.879	0.634	0.000	0.148	0.083	2.512	0.082	0.001	-0.863	0.082	-1.150	0.292	0.000

Supplementary Table 8. General linear model between brain-based sex score and cognitive GPS in European-ancestry participants Effects adjusted for covariates. *** P<0.001, ** P<0.01, * P<0.05

Cognitive GPS	Brain-based sex score in both group					Brain-based sex score in male group					Brain-based sex score in female group				
	β	SE	Y_{int}	P_{fdr}	η_p^2	β	SE	Y_{int}	P_{fdr}	η_p^2	β	SE	Y_{int}	P_{fdr}	η_p^2
Educational attainment	0.313	0.080	-3.387	0.000	0.007	0.200	0.114	-2.123	0.120	0.006	0.424	0.112	-4.020	0.001	0.014
Cognitive performance	0.349	0.094	-3.385	0.000	0.005	0.269	0.133	-2.019	0.120	0.007	0.432	0.133	-4.389	0.002	0.011
Intelligence quotient	-0.009	0.089	-1.717	0.921	0.001	0.149	0.132	-2.053	0.260	0.001	-0.143	0.122	-1.443	0.243	0.001

Supplementary Table 9. Orthogonalization regression between brain-based sex score and cognitive intelligence Effects adjusted for covariates. *** P<0.001, ** P<0.01, * P<0.05

Cognitive intelligence	Brain-based sex score in both group					Brain-based sex score in male group					Brain-based sex score in female group				
	β	SE	Y_{int}	P_{fdr}	η_p^2	β	SE	Y_{int}	P_{fdr}	η_p^2	β	SE	Y_{int}	P_{fdr}	η_p^2
Total intelligence	4320.724	387.289	25.805	0.000	0.016	2578.335	474.104	43.896	0.000	0.008	2978.230	306.961	14.100	0.000	0.025
Fluid intelligence	3691.094	490.313	39.844	0.000	0.007	1528.188	600.465	66.515	0.011	0.002	3002.295	387.705	18.832	0.000	0.016
Crystallized intelligence	3658.714	298.708	35.283	0.000	0.020	2853.268	362.014	39.463	0.000	0.016	2043.587	240.831	36.871	0.000	0.019

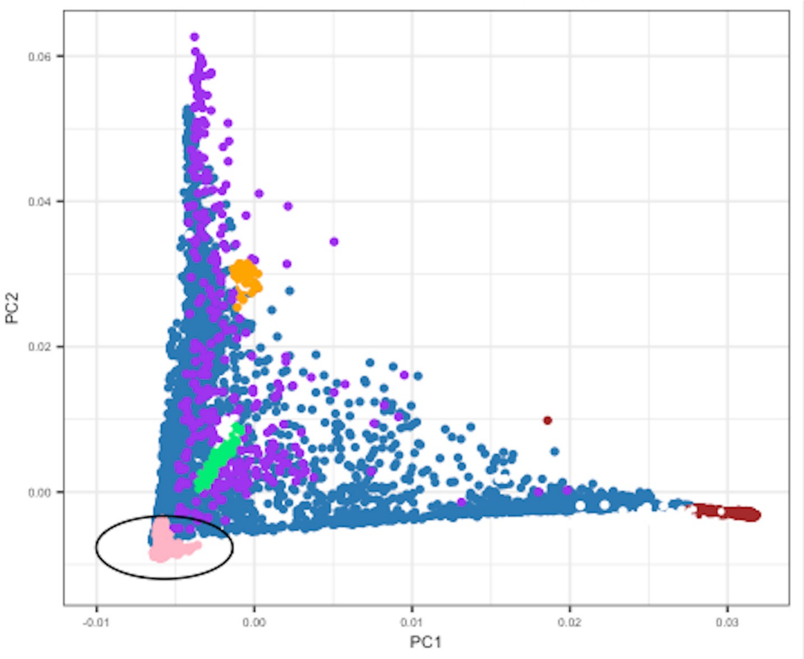

Supplementary Figure 1. PCA biplots of the genotype data of the study samples with 1000 Genomes phase3 reference samples. All the study samples were clustered as an admixed American population (super population code AMR), falling inside the mean pairwise Euclidean distance between admixed American samples. The color blue indicates ABCD subjects. The red-colored points represent AFR, purple-colored points represent AMR, green-colored points represent SAS, and orange-colored points represent EAS. The circled pink points indicate the European-ancestry individuals selected for ancestry specific sensitivity analysis. (AMR: Admixed American, AFR: African, EAS: East Asian, EUR: European; 1000 Genomes super population codes)

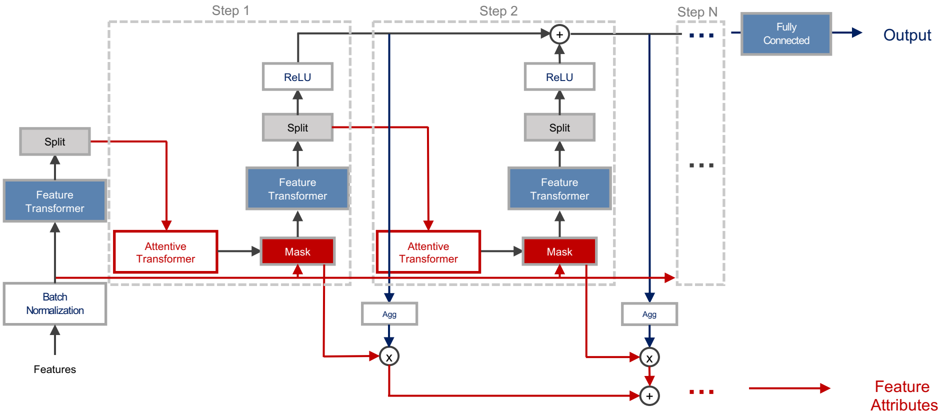

Supplementary Figure 2. Deep neural networks (DNN) architecture. TabNet: attentive interpretable tabular learning adapted from Arik and Pfister (2019).

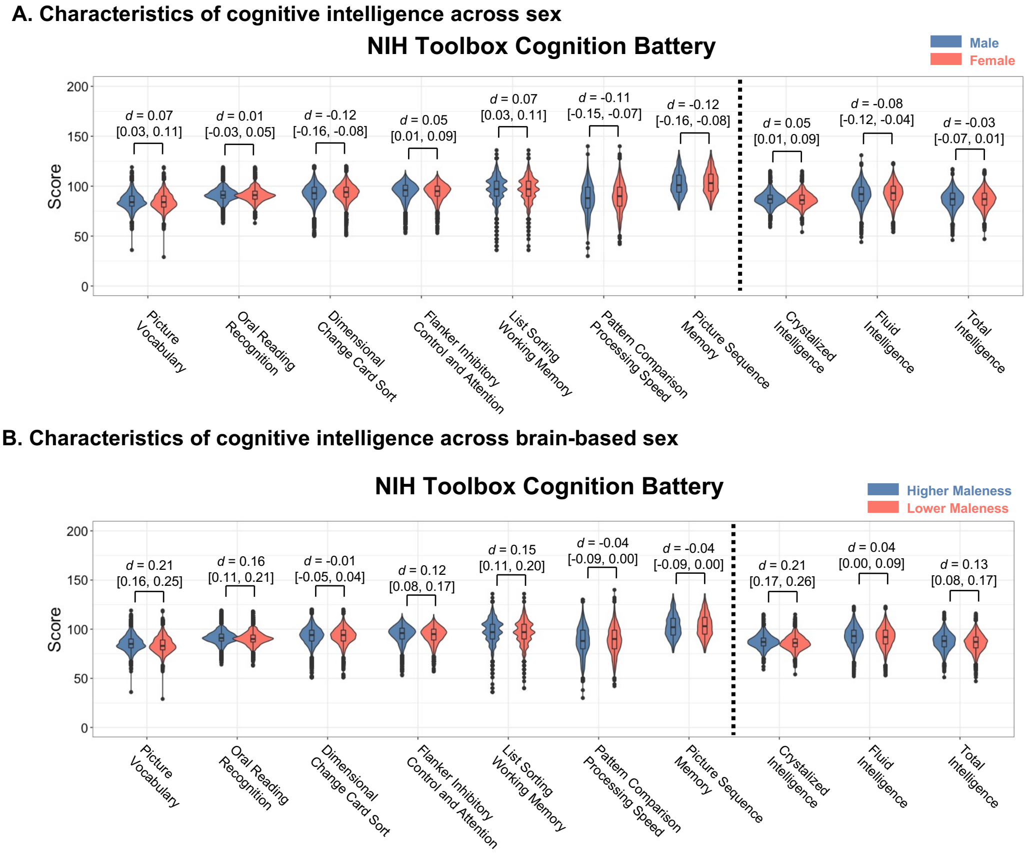

Supplementary Figure 3. Cognitive intelligence across sex and brain-based sex group. A, Characteristics of cognitive intelligence across sex. B, Characteristics of cognitive intelligence across brain-based sex. (brain-based maleness score >=0.5, higher maleness; <0.5, lower maleness) Group difference of cognitive intelligence between two groups were larger across brain-based sex group than biological sex group (Cohen's d < 0.21).

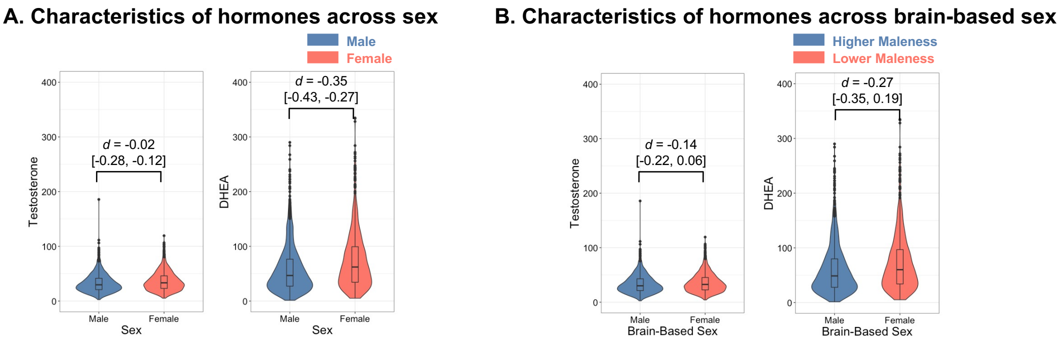

Supplementary Figure 4. **Characteristics of hormones across sex and brain-based sex.** A, Characteristics of hormones across sex. B, Characteristics of hormones across brain-based sex.

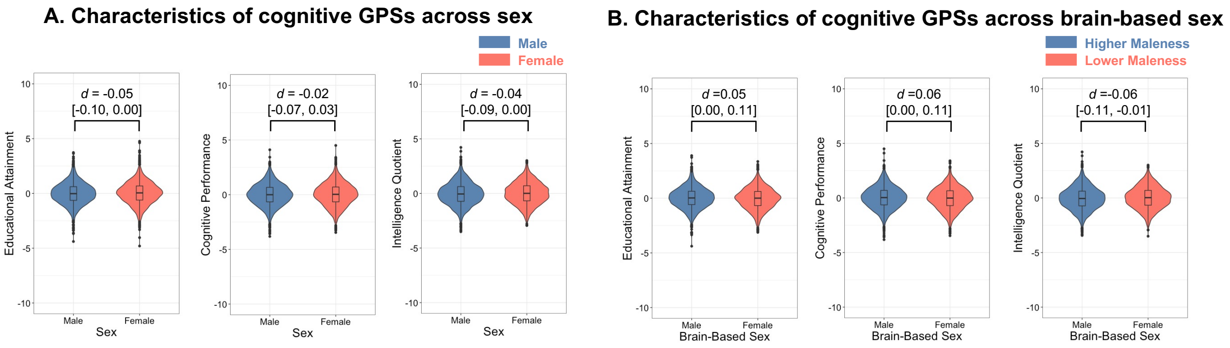

Supplementary Figure 5. Characteristics of cognitive GPSs across sex and brain-based sex. A, Characteristics of cognitive GPSs across sex. B, Characteristics of cognitive GPSs across brain-based sex.

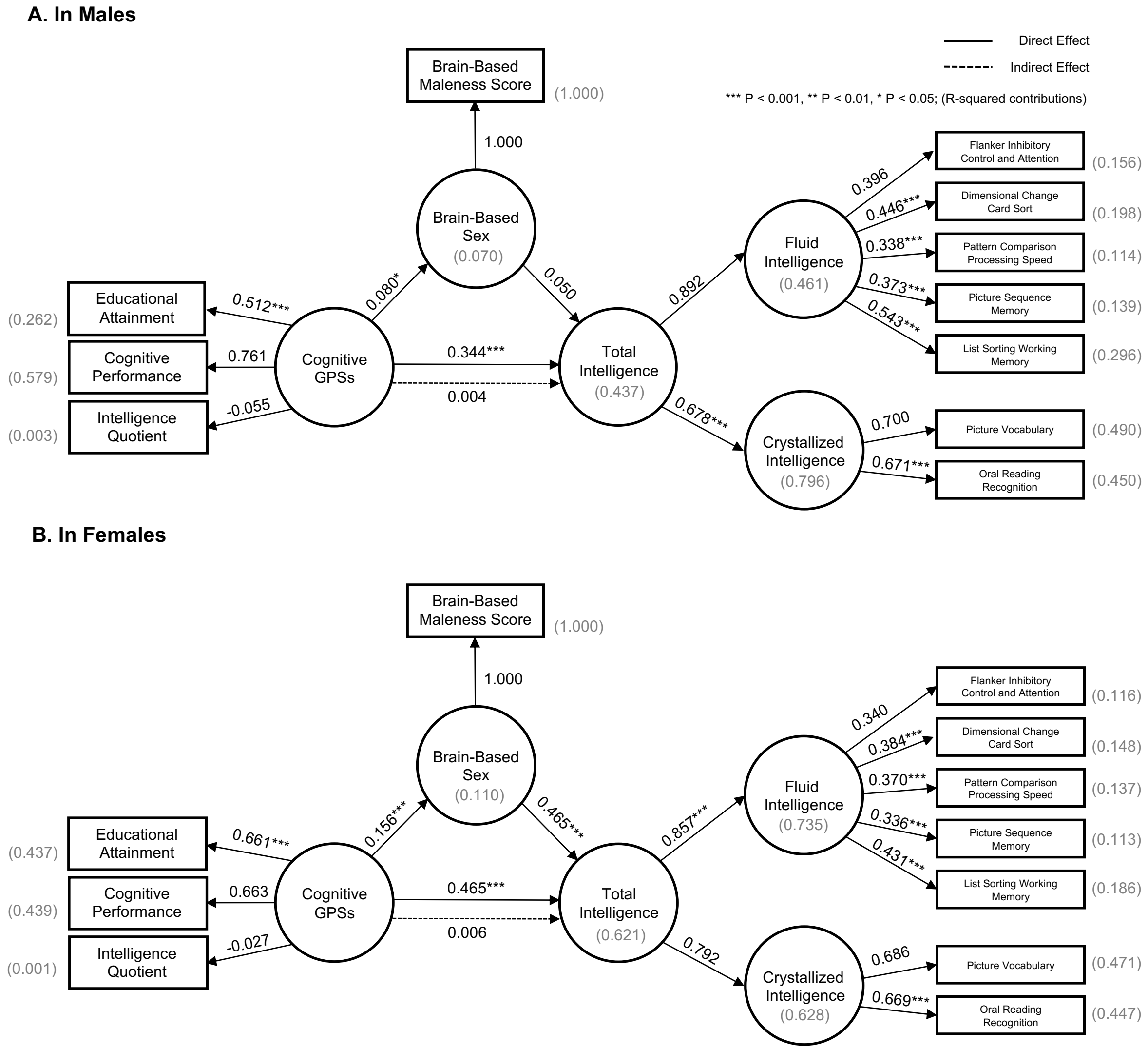

Supplementary Figure 6. Structural equation modeling of tripartite relationships: cognitive GPSs - brain-based sex score - cognitive intelligence in European-ancestry individuals. The direct effect is the pathway from the exogenous variable to the outcome while controlling for the mediator. The indirect effect is the pathway from the exogenous variable to the outcome through the mediator. A, Structural equation modeling of tripartite relationships in males. B, Structural equation modeling of tripartite relationships in females. Standardized weights are shown with statistical significance (bootstrapping). Shown in brackets are explained variances (R^2). *** <p.001

A. In Males

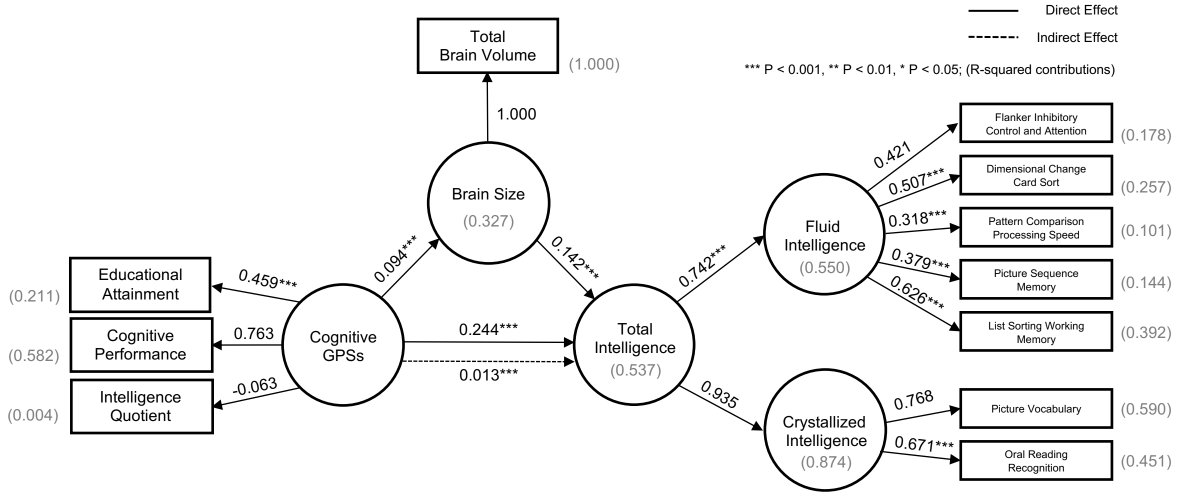

B. In Females

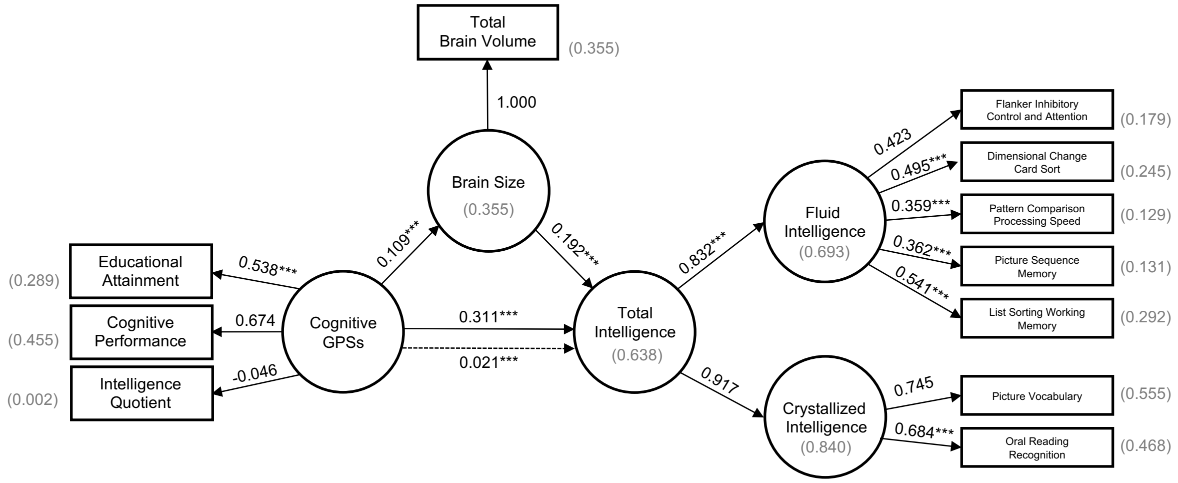

Supplementary Figure 7. Structural equation modeling of tripartite relationships: cognitive GPSs - brain size - cognitive intelligence. The direct effect is the pathway from the exogenous variable to the outcome while controlling for the mediator. The indirect effect is the pathway from the exogenous variable to the outcome through the mediator. A, Structural equation modeling of tripartite relationships in males. B, Structural equation modeling of tripartite relationships in females. Standardized weights are shown with statistical significance (bootstrapping). Shown in brackets are explained variances (R^2). *** <p.001
